# ETHNICITY, DEPRESSION, AND FUNCTIONAL OUTCOMES IN OLDER WOMEN

**DOI:** 10.1093/geroni/igz038.2253

**Published:** 2019-11-08

**Authors:** Cassandra M Germain

**Affiliations:** North Carolina A&T State University, Greensboro, North Carolina, United States

## Abstract

Women continue to have higher prevalence rates of functional impairment and depressive symptoms than men. In addition, women from certain ethnic groups experience disproportionately higher rates of ADL limitation, and are less likely to be screened for depression. The current study examines the association between race/ethnicity and depressive symptoms on functional limitations in Black, White and Hispanic women. We examined prevalence and adjusted odds of ADL limitations by race in n=9,846 women aged 50+ with low (CESD <4) and high (CESD =>4) depressive symptoms from the 2014 wave of the Health and Retirement Study. Overall, Black and Hispanic women had significantly higher rates of depression (p <.001) and ADL limitations (p<.001) than White women. Among those with high depressive symptoms, Black OR 1.98 [1.17, 3.34] and Hispanic OR 2.82 [1.42, 5.6] women have significantly higher rates of ADL limitations as compared to White women.

